# Cytochrome P450 reductase (POR) as a ferroptosis fuel

**DOI:** 10.1007/s13238-021-00823-0

**Published:** 2021-02-04

**Authors:** Pranavi Koppula, Li Zhuang, Boyi Gan

**Affiliations:** 1 Department of Experimental Radiation Oncology, The University of Texas MD Anderson Cancer Center, Houston, TX USA; 2 The University of Texas MD Anderson UTHealth Graduate School of Biomedical Sciences, Houston, TX USA

**Figure 1:**
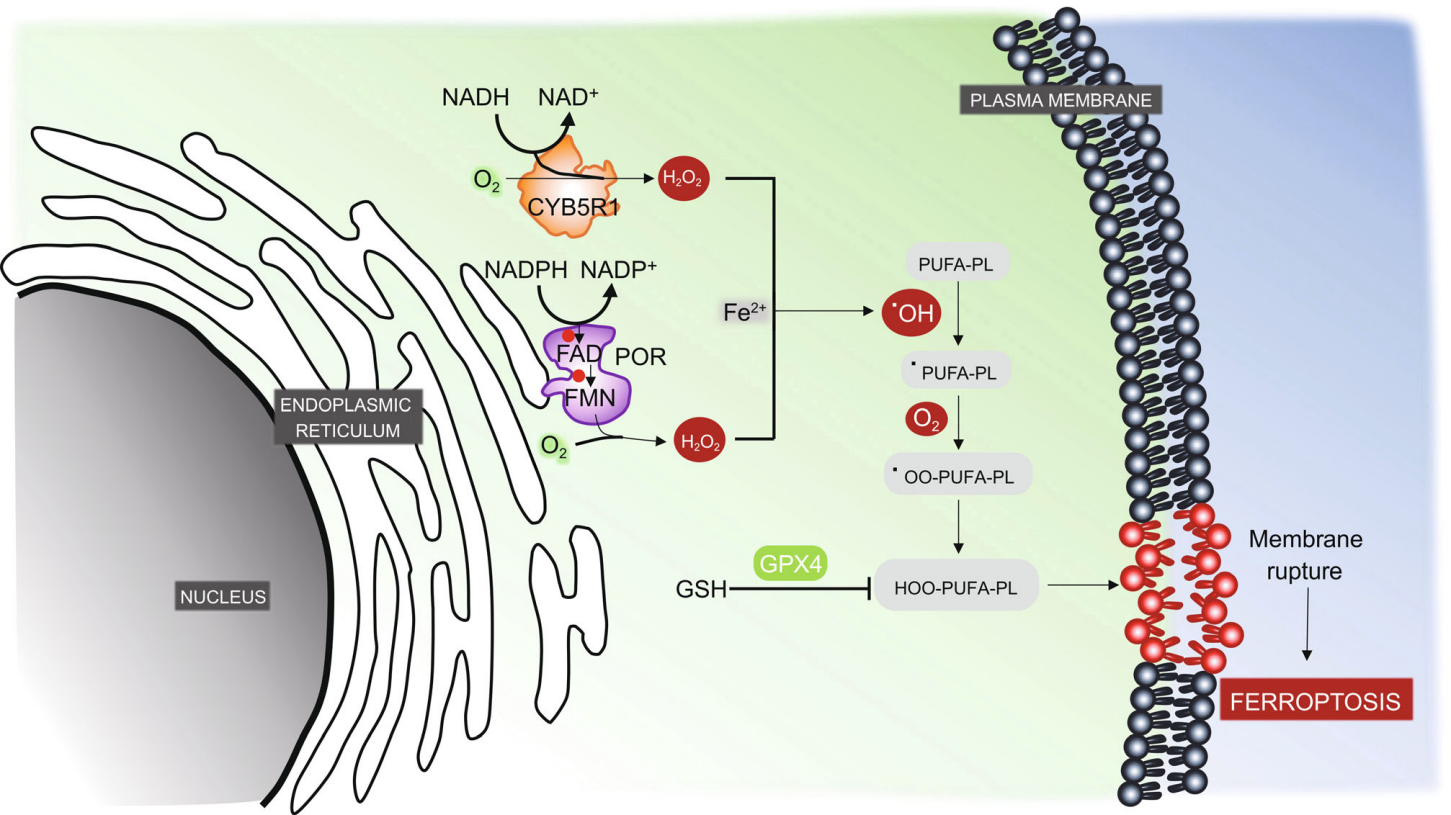
**POR and CYB5R1 promote lipid peroxidation and ferroptosis**. Oxidoreductases POR and CYB5R1 transfer electrons from NAD(P)H to O_2_ to generate H_2_O_2_, which then reacts with Fe^2+^ to drive lipid peroxidation. Lipid peroxides are detoxified by GPX4 and therefore are maintained at non-toxic levels under normal conditions. GPX4 inhibition leads to over-accumulation of lipid peroxides on cellular membranes, resulting in membrane rupture and eventually ferroptotic cell death. Of note, while POR is known to localize on the endoplasmic reticulum, the exact subcellular localization of CYB5R1 was not determined in the discussed studies. POR: cytochrome P450 reductase; CYB5R1: NADH-cytochrome b5 reductase 1; GPX4: glutathione peroxidase 4; GSH: glutathione; PUFA-PL: polyunsaturated fatty acid-containing phospholipid; ^•^PUFA-PL: PUFA-PL radicals; ^•^OO-PUFA-PLs: PUFA-PL peroxyl radicals; HOO-PUFA-PLs: PUFA-PL hydorperoxides; ^•^OH: hydroxyl radical; Fe^2+^: ferrous iron

Oxygen, iron, and polyunsaturated fatty acids (PUFAs; fatty acids containing more than one double bond) are all beneficial to our cellular lives. Incorporation of these components into cellular processes, however, comes at a cost: the bis-allylic structure of PUFAs and the enrichment of cellular environments with iron and oxygen render PUFA-containing phospholipids (PUFA-PLs) particularly susceptible to peroxidation (Yang and Stockwell, 2016). Accumulation of lethal amounts of lipid peroxides in cell membranes leads to a form of cell death known as ferroptosis (Dixon et al., 2012; Stockwell et al., 2017; Stockwell and Jiang, 2020). Consequently, cells are equipped with strong antioxidant defense systems that constantly dissipate toxic lipid peroxides generated in cellular membranes, thereby maintaining cell viability and homeostasis (Zheng and Conrad, 2020). The most powerful anti-ferroptosis defense system is believed to be mediated by glutathione peroxidase 4 (GPX4), a glutathione peroxidase that uses glutathione as its cofactor to reduce lipid hydroperoxides to non-toxic lipid alcohols (Fig. 1) (Zheng and Conrad, 2020). A variety of ferroptosis inducers (FINs) act to inactivate GPX4 or deplete glutathione, causing an imbalance between the production and detoxification of lipid peroxides that subsequently induces ferroptotic cell death (Yang et al., 2014). Genetic ablation of GPX4 can have the same effect (Friedmann Angeli et al., 2014).

Exactly how lipid peroxidation occurs in PUFA-PLs is less well understood, but lipid peroxidation is known to involve both non-enzymatic reactions (e.g., autoxidation, which requires iron and oxygen) and protein enzymes (Yin et al., 2011; Conrad and Pratt, 2019). Enzymatic lipid peroxidation was initially thought to be carried out mostly by lipoxygenases (ALOXs) (Yang et al., 2016; Wenzel et al., 2017). However, several lines of evidence from recent studies argue against ALOXs being essential drivers of lipid peroxidation. First, the ALOX inhibitors that were used to establish the role of ALOX in regulating ferroptosis turned out to also harbor a previously unrecognized radical-trapping antioxidant activity (Shah et al., 2018). Therefore, whether these compounds block ferroptosis by inhibiting ALOXs or by trapping lipid peroxyl radicals remains unclear. Further, ALOXs are expressed in extremely low levels in most cancer cell lines (Zou et al., 2020b), raising further questions about its role in regulating ferroptosis in broad contexts. Finally, depleting ALOXs in cancer cells that already have low ALOX expression did not affect the cells’ sensitivity to ferroptosis (Zou et al., 2020b). These findings suggest that one or more additional enzymes exist to mediate lipid peroxidation, the identity of which was revealed in two recent studies (Yan et al., 2020; Zou et al., 2020b).

In both studies, CRISPR screening identified two genes, acyl-coenzyme A (CoA)-synthetase long-chain family member 4 (*ACSL4*) and cytochrome P450 reductase (*POR*), as top suppressor hits (whose inactivation suppressed FIN-induced ferroptosis, resulting in relative enrichment of their gRNAs in FIN-treated cells relative to that in control cells). ACSL4 is an acyl-CoA synthetase that converts free long-chain fatty acids into fatty acyl-CoA esters, with a preference for PUFAs. *ACSL4* deficiency is known to compromise PUFA-PL biosynthesis, leading to remarkable ferroptosis resistance in cells (Dixon et al., 2015; Doll et al., 2017; Kagan et al., 2017). Therefore, the identification of ACSL4 as a top suppressor hit validated these CRISPR screens. However, the potential role of POR in ferroptosis had been previously unknown. *POR* deletion was subsequently confirmed to promote strong resistance to ferroptosis induced by different FINs and in a wide range of cancer cell lines. *POR* deficiency did not affect levels of GPX4 or its cofactor glutathione, binding between GPX4 and its inhibitors, or phospholipid profiles, but *POR* deficiency significantly reduced the levels of peroxidized PL species resulting from treatment with FINs, indicating that POR promotes lipid peroxidation during ferroptotic stress (Yan et al., 2020; Zou et al., 2020b). Notably, POR is expressed at high levels in most cancer cell lines (Zou et al., 2020b), supporting its role as a general driver of lipid peroxidation.

These findings prompt the question of how POR governs the production of lipid peroxides. POR is an oxidoreductase located in the endoplasmic reticulum that uses FAD and FMN as its cofactors to donate electrons from NADPH to cytochrome P450 (CYPs) as well as other proteins, and it participates in controlling xenobiotic detoxification and redox homeostasis (Pandey and Fluck, 2013). Re-expression of wild-type POR, but not its mutants with defective electron transfer activity, in *POR*-deficient cells restored ferroptosis sensitivity, indicating that the electron transfer activity of POR is required for its function in ferroptosis regulation (Yan et al., 2020). Zou et al. speculated that by donating electrons to CYPs, POR might facilitate Fenton reactions in the heme component of CYPs and thereby lipid peroxidation (because Fenton reactions are required for autoxidation during lipid peroxidation) (Zou et al., 2020b). Yan et al. showed that deletion of the transmembrane region in POR that mediates the interaction of POR with CYPs abolishes the ability of POR to donate electrons to CYPs, yet this mutant acts like its wild-type counterpart in terms of promoting lipid peroxidation and ferroptosis, indicating that POR regulates lipid peroxidation in a manner independent of CYPs (Yan et al., 2020). Further, deletion of other POR electron acceptor proteins, such as heme oxygenase, cytochrome squalene monooxygenase, or cytochrome b5, did not affect ferroptosis sensitivity, suggesting that these other proteins downstream of POR also do not mediate the function of POR in ferroptosis regulation (Yan et al., 2020). However, because there exist multiple CYP members and other proteins that can act as electron acceptors downstream of POR in a highly redundant manner, their roles in mediating POR’s function in regulating ferroptosis cannot be completely excluded and require further investigations (such as by compound knockout).

Noting that the ferroptosis-resistance phenotype in *POR*-deficient cells is milder than that in cells treated with ferroptosis inhibitors, Yan et al. reasoned that other oxidoreductases may operate in parallel with POR to mediate lipid peroxidation and ferroptosis. This led to the identification of NADH-cytochrome b5 reductase 1 (CYB5R1) as yet another oxidoreductase whose deficiency suppresses lipid peroxidation and ferroptosis, albeit with more moderate effects than POR inactivation; combined deficiency of both *POR* and *CYB5R1* resulted in even more pronounced ferroptosis resistance than did deficiency of either gene alone (Yan et al., 2020). It is possible that CYB5R1 acts as a context-specific contributor to ferroptosis, and other unidentified oxidoreductases may be involved in regulating ferroptosis under other cellular contexts.

Because ferroptosis is a form of oxidative stress-induced cell death, and both POR and CYB5R1 have roles in redox regulation, Yan et al. further reasoned that POR and CYB5R1’s function in governing lipid peroxidation and ferroptosis could relate to their potential abilities to generate reactive oxygen species, likely through electron transfer. In support of this hypothesis, they showed that purified POR could produce H_2_O_2_ (but not superoxide) in an NADPH- and oxygen-dependent manner and that *POR*-deficient cells exhibit lower levels of intracellular H_2_O_2_ (Yan et al., 2020). CYB5R1 had similar, albeit more moderate effects, which is consistent with the effects of these two proteins in inducing lipid peroxidation and ferroptosis. Moreover, treatment with H_2_O_2_ re-sensitized *POR*-deficient cells (which are resistant to ferroptosis) to ferroptosis. This series of elegant experiments strongly suggests that POR (as well as CYB5R1) promotes lipid peroxidation and ferroptosis through the generation of H_2_O_2_ (Fig. 1). H_2_O_2_ can participate in lipid peroxidation probably through the following reactions (Conrad and Pratt, 2019): H_2_O_2_ is first converted to hydroxyl radicals (^•^OH) via its reaction with Fe^2+^. The ^•^OH can subsequently abstract a hydrogen from the bis-allylic moieties in PUFA-PLs, resulting in PUFA-PL radicals (^•^PUFA-PLs), which then react with oxygen to produce PUFA-PL peroxyl radicals (^•^OO-PUFA-PLs) and PUFA-PL hydroperoxides (HOO-PUFA-PLs) (Fig. 1).

Exactly how lipid peroxidation eventually triggers ferroptotic cell death remains a central question (Stockwell et al., 2020). Thus far, two non-mutually exclusive models have been developed to address this question. In one model, lipid peroxidation is thought to damage cellular membranes directly, leading to membrane rupture and cell demise; in the other model, lipid peroxidation is thought to trigger downstream signaling events culminating in the activation of a cell-death “executioner” that can perforate cellular membranes and kill cells, much like MLKL in necroptosis or gasdermins in pyroptosis (Galluzzi et al., 2018). (This latter model would predict that, unlike *ACSL4* or *POR* deficiency, inactivation of such a ferroptosis executioner should not affect FIN-induced lipid peroxidation but would still drive ferroptosis resistance, thereby uncoupling lipid peroxidation from ferroptotic cell death. However, no gene with this characteristic has been identified so far.) In a remarkable experiment, Yan and colleagues demonstrated that purified recombinant POR, together with iron and NADPH, caused significant leakage from PUFA-containing liposomes in a cell-free system, accompanied by liposomal rupture; this POR-induced liposome leakage could be rescued by treatment with a ferroptosis inhibitor (Yan et al., 2020). These analyses provide compelling evidence that POR-catalyzed lipid peroxidation is sufficient to induce membrane rupture.

To place their findings in the context of ferroptosis-associated diseases, Yan et al. studied the potential role of POR in Concanavalin A (ConA)-induced acute liver injury, which has been causally linked with ferroptosis (Zeng et al., 2020). POR depletion in mouse liver markedly suppressed ConA-induced liver damage and animal lethality; likewise, ConA treatment induced higher levels of the ferroptosis markers *PTGS2* and malondialdehydes in mouse liver, whereas POR depletion attenuated these ConA-induced ferroptosis markers, indicating that POR is required for ConA-induced acute liver injury, probably by inducing lipid peroxidation and ferroptosis *in vivo* (Yan et al., 2020).

Yan et al. further suggested that POR could be considered an “executioner” for ferroptosis, as it likely to be the protein involved in the final step in triggering ferroptotic cell death. It should be noted that there are several notable differences between POR and other classic cell death executioners such as MLKL and gasdermins. First, unlike MLKL and gasdermins, POR does not have the pore-forming activity. In addition, cell death modalities, such as apoptosis, necroptosis, and pyroptosis, are considered cell “suicide” programs that utilize evolutionarily conserved developmental pathways to actively clear unwanted cells in an organism and are triggered by dedicated cell death executioner proteins, whose activation is often governed by upstream signaling. In contrast, ferroptosis is viewed as a cell “sabotage” program, wherein the cell death results from metabolic imbalance that goes beyond the control of cellular antioxidant buffering systems (Green and Victor, 2012). As such, it appears that the pro-ferroptosis factors identified so far are not proteins dedicated in cell death regulation, but rather those incidentally involved in generating ferroptosis-triggering metabolites in a constitutive fashion. For example, ACSL4’s professional function is to regulate PUFA-PL biosynthesis. Because PUFA-PLs are susceptible to peroxidation, ACSL4 appears to participate in ferroptosis induction in a more “incidental” manner. The same principle can be applied to the role of POR in triggering ferroptosis. For these considerations, whether POR can be classified as a cell death executioner is certainly debatable.

Together, these two recent studies identified POR as a key player to “fuel” ferroptosis. Mechanistically, POR (and CYB5R1) can transfer electrons from NAD(P)H to oxygen to produce H_2_O_2_, which then drives lipid peroxidation, membrane rupture, and ferroptosis (Fig. 1). These intriguing studies also raised several important questions. The exact roles of H_2_O_2_ (and metabolic enzymes involved in H_2_O_2_ production or detoxification) in ferroptosis regulation remain to be further studied. A previous study showed that replacement of the selenocysteine residue in GPX4 with cysteine (*GPX4*^*cys*/*cys*^ mutant) rendered cells exquisitely sensitive to H_2_O_2_-induced cell death; interestingly, while H_2_O_2_-induced cell death in *GPX4*^*cys*/*cys*^ cells could be rescued by ferroptosis inhibitors, these inhibitors failed to rescue cell death induced by H_2_O_2_ at much higher concentrations in wild-type counterparts (Ingold et al., 2018). Therefore, it appears that H_2_O_2_ induces ferroptosis in a context- and dose-dependent manner. Further, while overexpression of a genetically engineered cytosol-localized catalase (an enzyme that normally localizes in peroxisomes and catalyzes H_2_O_2_ decomposition in peroxisomes) in *POR*-proficient cells promoted ferroptosis resistance (Yan et al., 2020), another recent study showed that deleting endogenous catalase (and thereby abolishing H_2_O_2_ decomposition in peroxisomes) did not affect ferroptosis sensitivity in other cancer cell lines (Zou et al., 2020a), suggesting a cellular compartment- or cell line-dependent role of catalase in ferroptosis protection. The exact role of catalase in ferroptosis protection therefore remains to be studied in broader contexts. Finally, given the association of ferroptosis with diverse diseases and conditions such as cancer, ischemia/reperfusion-induced organ damage, and degenerative diseases (Gao et al., 2015; Jiang et al., 2015; Stockwell et al., 2017; Zhang et al., 2018; Lee et al., 2020; Zheng and Conrad, 2020), future investigations should also be directed to understanding the role of POR in these other ferroptosis-associated diseases and the potential applications of POR inhibitors for treating diseases caused by excessive ferroptosis.
